# EMT-Induced Stemness and Tumorigenicity Are Fueled by the EGFR/Ras Pathway

**DOI:** 10.1371/journal.pone.0070427

**Published:** 2013-08-12

**Authors:** Dominic Chih-Cheng Voon, Huajing Wang, Jason Kin Wai Koo, Juin Hsien Chai, Yit Teng Hor, Tuan Zea Tan, Yeh-Shiu Chu, Seiichi Mori, Yoshiaki Ito

**Affiliations:** 1 The Cancer Science Institute of Singapore, National University of Singapore, Singapore, Singapore; 2 Brain Research Center, National Yang-Ming University, Taipei, Republic of China; 3 Division of Cancer Genomics, Cancer Institute of Japanese Foundation for Cancer Research, Tokyo, Japan; 4 The Institute of Molecular and Cell Biology, Agency for Science, Technology and Research, Singapore, Singapore; University of Colorado, Boulder, United States of America

## Abstract

Recent studies have revealed that differentiated epithelial cells would acquire stem cell-like and tumorigenic properties following an Epithelial-Mesenchymal Transition (EMT). However, the signaling pathways that participate in this novel mechanism of tumorigenesis have not been fully characterized. In *Runx3* ^−/−^ *p53* ^−/−^ murine gastric epithelial (GIF-14) cells, EMT-induced plasticity is reflected in the expression of the embryonal proto-oncogene *Hmga2* and *Lgr5*, an exclusive gastrointestinal stem cell marker. Here, we report the concurrent activation of an EGFR/Ras gene expression signature during TGF-β1-induced EMT in GIF-14 cells. Amongst the altered genes was the induction of *Egfr*, which corresponded with a delayed sensitization to EGF treatment in GIF-14. Co-treatment with TGF-β1 and EGF or the expression of exogenous KRas led to increased *Hmga2* or *Lgr5* expression, sphere initiation and colony formation in soft agar assay. Interestingly, the gain in cellular plasticity/tumorigenicity was not accompanied by increased EMT. This uncoupling of EMT and the induction of plasticity reveals an involvement of distinct signaling cues, whereby the EGFR/Ras pathway specifically promotes stemness and tumorigenicity in EMT-altered GIF-14 cells. These data show that the EGFR/Ras pathway requisite for the sustenance of gastric stem cells *in vivo* and *in vitro* is involved in the genesis and promotion of EMT-induced tumor-initiating cells.

## Introduction

Epithelial-Mesenchymal Transition (EMT) is a developmental program that plays an instrumental role in early embryo patterning during gastrulation [1]. During EMT, epithelial cells are temporarily reprogrammed to lose their defining features such as cell-cell adhesion, epithelial tight junction and desmosomes. Concurrently, there is a gain of mesenchymal properties, including increased cell migration and resistance to anoikis. These profound changes reflect a highly coordinated genetic reprogramming effected by specialized transcription factors, such as Snail, Twist and Zeb, that are activated in response to extracellular cues, most notably Transforming Growth Factor beta (TGF-β) [1].

TGF-β is a pleiotropic growth factor that also mediates tumor suppressive effects in multiple adult tissues. Components of the TGF-β pathway are frequently targeted by mutations in human carcinomas [2]. However, in advanced cancer the TGF-β pathway is paradoxically a major driver of tumor progression and metastasis due in part to its aberrant activation of EMT [1]. More recently, evidence have emerged that the aberrant induction of EMT endows cellular plasticity and stem-like properties in differentiated mammary epithelial cells, giving rise to so-called cancer stem cells [3], [4]. Intriguingly, these metastable mesenchymal and stem cell-like states could be established solely by paracrinal and autocrinal signals, specifically the TGF-β and the canonical and non-canonical Wnt pathways [5]. Notably, these pathways feature prominently in the self-renewal of the mammary epithelium, implicating a common mechanism in maintaining the epigenetic states of normal and cancer stem cells.

In the gastrointestinal epithelium, the stem cells at the base of the pyloric gastric glands and intestinal crypts are similarly reliant on an active and dynamically regulated Wnt pathway [6], [7]. This dependency is reflected in the exclusive expression of Lgr5, which functions to amplify the Wnt signal in these stem cells [8], [9]. In addition to Wnt, a delicate balance of BMP, Notch and Epidermal Growth Factor (EGF) signaling within the intestinal stem cell niche is crucial to the maintenance of the stem cell state [10]–[14]. During injury, modulation of the Wnt signal would induce a state of plasticity in a specific subset of progenitor cells, enabling their dedifferentiation to replace damaged Lgr5^+ve^ stem cells [15]. The induction of a stem cell state in differentiated cells in response to damage and increased Wnt signal in the intestinal crypt parallels the aforementioned observations in mammary epithelial cells, which together suggest a role for induced plasticity under physiological conditions and during carcinogenesis. This is supported by the participation of Lgr5 in supporting Wnt-driven intestinal adenomas in mouse, and cancer stem cells isolated from primary human colon tumors [16], [17].

In a previous study, we observed in an immortalized *Runx3* ^−/−^ *p53* ^−/−^ gastric epithelial (GIF-14) cell line a stem-like and tumorigenic subpopulation that arose from spontaneous EMT [18]. The acquisition of cellular plasticity following EMT is reflected in the elevated expression of stem cell-associated markers *Lgr5*, *Sox9* and *Hmga2*. In contrast, *Runx3* ^+/+^ *p53* ^−/−^ cells are resistant to EMT, demonstrating a safeguarding role for Runx3, which is an established gastric tumor suppressor [19], [20]. A primary driver of EMT in GIF-14 is the TGF-β pathway, as treatment with exogenous TGF-β1 or pharmacological inhibition of TGF-βRI readily induced interchangeable mesenchymal- and epithelial-like states, respectively [18]. Here, we demonstrate that while the TGF-β pathway is crucial in initiating EMT, other physiologically relevant pathways participate in promoting plasticity and a stem cell-like state in GIF-14 cells. In particular, we observed the activation of an EGFR/Ras gene expression signature during TGF-β1-induced EMT in GIF-14 cells, which included increased *Egfr* expression. This endowed GIF-14 cells increased responsiveness to EGF, which acted in concert with TGF-β1 to activate *Hmga2* expression. Consistent with this cooperation, pharmacological inhibition of MEK, a downstream effector of EGFR, effectively blocked TGF-β1-activated *Hmga2* expression. A functional contribution of the Ras pathway to stemness and tumorigenicity of GIF-14 cells was further demonstrated in the increased sphere initiation and colony formation in response to exogenous KRas. Surprisingly, the KRas-induced stemness and tumorigenicity were not accompanied by increased EMT in GIF-14. Together, these data reveal a novel relationship between two physiologically important signals in the induction and maintenance of a stem-like state in gastric epithelial cells.

## Results

### An EGFR/RAS gene expression signature corresponds with TGF-β1-induced EMT in GIF-14 cells

**Figure 1:**
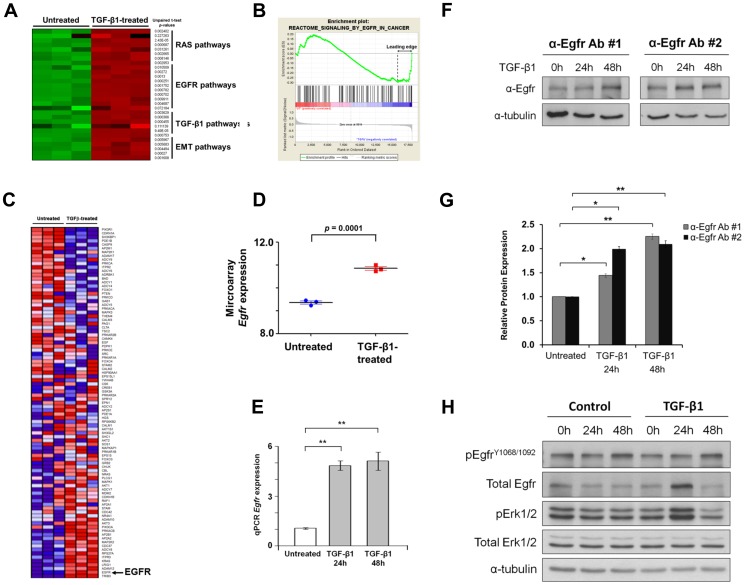
TGF-β1 induces EGFR/Ras gene expression signature. (A) A heat map highlighting the results of gene set enrichment analysis of genes significantly altered by TGF-β1 (2.5 ng/ml) in GIF-14 cells (nominal *p*-values<0.05). Mean gene expression value of leading-edge genes of each gene set is plotted. Lower levels of expression are represented in green and higher expression in red. Gene sets representing differentially enriched pathways are grouped. (B) The Enrichment Plot of a representative EGFR gene set. The relative gene positions of gene set are indicated by the vertical lines (middle) under the graph, which presents the enrichment scores of individual genes (top). Lines clustered to the left represent higher ranked genes in the ranked list. Bottom plot displays the rank matrix of these genes. The position of leading-edge genes suggests a positive correlation between TGF-β1 treatment and EGFR pathway. (C) A heat map depicting the expression levels of genes within the EGFR gene set in response to 24 h of TGF-β1 treatment, as measured by expression microarray analysis. Each column represents the expression data derived from a single replicate (n = 3). Gene expression is normalized for each row; where lower levels of expression are represented in shades of blue and higher expression in red. The arrow highlights an induction of *Egfr* mRNA in response to TGF-β1. (D) Normalized *Egfr* mRNA expression level as measured by microarray. (E) Validation of TGF-β1 induction of *Egfr* expression by quantitative PCR. Expression of *Egfr* are normalized against that of *Gapdh* and expressed relative to the untreated control (means ± SEM, n = 4). Student's t-tests are performed and double asterisks represent *p*-value<0.01. (F) Western blot analysis of total Egfr expression as measured by two separate Egfr-specific antibodies – Ab #1 (Abcam) and Ab #2 (Cell signaling). The expression level of α-tubulin is used as a control for the amounts of protein lysates loaded. (G) Protein band intensities are assessed by densitometric analysis. The band intensities of Egfr in Figure 1F were sampled three times and normalized against that of α-tubulin (means ± SEM, n = 3). Student's t-tests are performed in which single and double asterisks denote *p* value<0.05 and *p* value<0.01, respectively. (H) Western blot analysis of phosphorylated Egfr and Erk expression in response to TGF-β1 treatment. GIF-14 cells were treated with TGF-β1 (2.5 ng/ml) for 24 h and 48 h before harvesting for Western blotting. Phosphorylation of Egfr at tyrosine residues 1068 and 1092 and Erk1/2 was detected by pEgfr^Y1068/1092^- and pErk1/2–specific antibodies. Total Egfr expression levels were measured using anti-Egfr antibody #2 as shown in Figure 1F. Immunoblots of α-tubulin serves as a control for the amount of proteins loaded.

In a previous study, it was observed that GIF-14 cells readily undergo EMT upon treatment with TGF-β1, giving rise to a tumorigenic, stem-like subpopulation. To elucidate the gene expression changes that precede this phenomenon, the pre-EMT, epithelial subpopulation of GIF-14 cells was fractionated and treated with TGF-β1 and subjected to expression microarray analyses. These revealed that 2135 genes were significantly altered within 24 h post-TGF-β1 treatment. In addition to changes in genes associated with TGF-β and EMT, Gene Set Enrichment Analysis (GSEA) revealed a significant enrichment of gene sets belonging to the Epidermal Growth Factor Receptor (EGFR) and RAS pathways (Figure 1A). Amongst the most prominent leading-edge genes enriched within the EGFR/RAS gene sets were *Egfr*, *KRas* and *Lrig1* (Figure 1B & 1C). Egfr and KRas are molecular switches responsible for the activation of EGFR/RAS pathways, while Lrig1 is a pan-negative regulator of ErbB pathways, including EGFR pathway [21]. Perturbation of the expression of these genes by TGF-β1 treatment suggests a cross-talk between TGF-β1 and EGFR/RAS pathways. The induction of *Egfr* levels by TGF-β1 treatment observed in the microarray analyses was subsequently confirmed by qRT-PCR, which showed that *Egfr* expression was induced in GIF-14 cells within 24 h of TGF-β1 treatment and persisted for at least 48 h (Figure 1D & 1E). To further validate this at the level of protein expression, Western blotting of Egfr protein was performed on TGF-β1-treated GIF-14 cells using two independent anti-Egfr antibodies (Figure 1F). Although the increase in Egfr protein expression in this experiment is relatively modest, densitometric measurement of band intensities confirm the induction of Egfr expression by TGF-β1 (Figure 1G). To determine if the increased Egfr expression translates into an activation of the EGFR/Ras signaling pathway, the phosphorylation of Egfr at tyrosine residues 1068 and 1092 and Extracellular signal-Regulated Kinase (Erk) 1/2 were measured by Western blotting using pEgfr^Y1068/1092^- and pErk1/2 -specific antibodies. This revealed a transient induction of Egfr within 24 h of TGF-β1 treatment, which was accompanied by a clear increase of pErk1/2. However, the level of pEgfr^Y1068/1092^ remained unchanged, suggesting the involvement of an alternative mechanism that is independent of phosphorylation at the two residues studied (Figure 1H). Interestingly, while the induction of Egfr expression was robust in this experiment, it was not sustained as that shown in Figure 1F, indicating that Egfr is tightly regulated by negative feedback mechanisms, such as that involving Lrig1 [21].

### TGF-β1 cooperates with EGF in inducing stemness in GIF-14 cells

**Figure 2:**
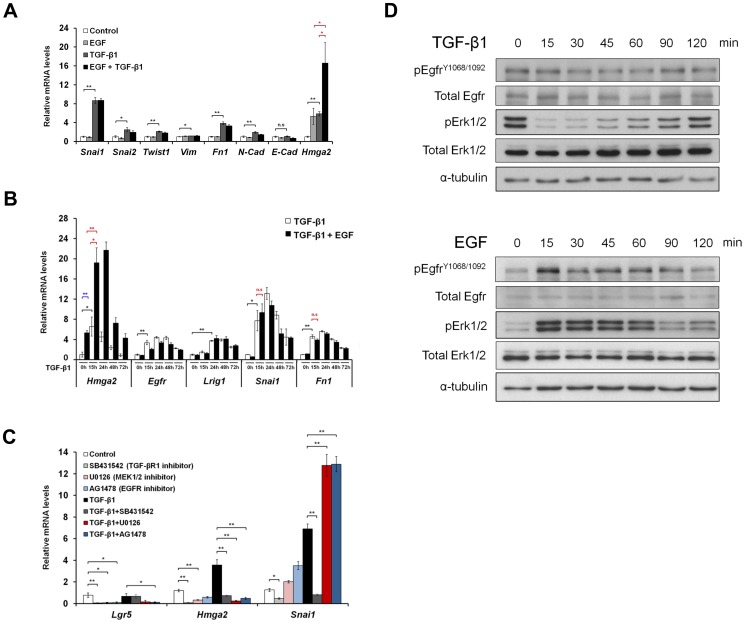
TGF-β and EGFR pathways cooperate to induce stemness in GIF-14 cells. (A) Changes in the expression of stemness- and EMT/mesenchymal-associated marker genes in response to TGF-β1 and EGF. GIF-14 cells were treated with murine EGF (10 ng/ml) or TGF-β1 (2.5 ng/ml) or in combination for 15 h. Quantitative PCR measurements of gene expression levels are normalized against *Gapdh* levels, and expressed relative to the control sample (means ± SEM, n = 4). Student's t-tests are performed in which single and double asterisks denote *p* value<0.05 and *p* value<0.01, respectively and n.s represents not significant (Black bracket: TGF-β1 responsiveness; Red bracket: cooperative induction by TGF-β1 and EGF). (B) Cooperative induction of stemness by EGF and TGF-β1. GIF-14 cells were pretreated with TGF-β1 (2.5 ng/ml) for varying periods at 15 h, 24 h, 48 h and 72 h before the addition of murine EGF (10 ng/ml) for another 15 h. Changes in the mRNA levels of stemness marker *Hmga2*, regulators of EGF signaling *EGFR* and *Lrig1*, EMT markers *Snai1* and *fibronectin1* (*Fn1*) were determined by qRT-PCR. The values are normalized against those of *Gapdh* and are expressed relative to that of the control (means ± SEM, n = 3). Student's t-tests are performed where indicated. Single and double asterisks represent *p* value<0.05 and *p* value<0.01, respectively while n.s denotes not significant (Black bracket: TGF-β1 responsiveness; Blue bracket: EGF responsiveness; Red bracket: cooperative induction by TGF-β1 and EGF). (C) TGF-β1 induction of *Hmga2* is abrogated by inhibitors of EGFR and MEK1/2. GIF-14 cells were treated with SB431542 (TGF-βRI inhibitor; 10 µM) or AG1478 (EGFR inhibitor; 10 µM) or U0126 (MEK1/2 inhibitor; 10 µM) or in combination with TGF-β1 (2.5 ng/ml) for 48 h. Changes in the mRNA levels of stemness marker *Hmga2* and EMT marker *Snai1* were ascertained by qRT-PCR and normalized values are expressed relative to the control values (means ± SEM, n = 4). Student's t-tests are performed where indicated. Single and double asterisks denote *p* value<0.05 and *p* value<0.01, respectively. (D) The effects of TGF-β1 and EGF on the phosphorylation states of Egfr and Erk. GIF-14 cells were treated with TGF-β1 (2.5 ng/ml; top panel) or murine EGF (10 ng/ml; bottom panel) for various short periods of time from 15 to 120 min. The expression levels of phosphorylated Egfr at tyrosine residues 1068 and 1092 and Erk1/2 were measured by Western blot analysis using pEgfr^Y1068/1092^- and pErk1/2–specific antibodies Total Egfr expression was determined using anti-Egfr antibody #2 as shown in Figure 1F. The expression level of α-tubulin serves as a control for the amount of proteins loaded.

In GIF-14 cells, the entrance into EMT is marked by increased expression of EMT-inducing transcription factors *Snai*1, *Snai*2 and *Twist1*; mesenchymal markers *vimentin* (*Vim*), *fibronectin1* (*Fn1*) and N-cadherin (N-cad); and stemness markers *Lgr5* and *Hmga2* [18]. To investigate the involvement of EGFR and other signaling pathways known to induce EMT, GIF-14 cells were treated with EGF, Fibroblast Growth Factors (FGF) 2 and 10, and Hepatocyte Growth Factor (HGF) and TGF-β1. Changes in the expression of EMT/Stemness gene signature were measured by qRT-PCR. The results confirm that TGF-β1 is a major driver of EMT in GIF-14 cells while the other growth factors did not promote EMT (Figure S1). However, EGF further augmented TGF-β1's induction of *Hmga2* a marker of stemness and plasticity [22]–[24] (Figure S1). To investigate this further, GIF-14 cells were treated with EGF and TGF-β1 separately or in combination for 15 h. Gene expression analysis confirms the cooperation between TGF-β1 and EGF on the induction of *Hmga2*. However, EGF did not cooperate with TGF-β1 on the induction of all EMT markers tested (Figure 2A).

As the induction of Egfr occurred after 24 h of TGF-β1 treatment, we next sought to determine if the observed cooperation between TGF-β1 and EGF is dependent on the duration of TGF-β1 treatment. To do this, GIF-14 cells were subjected to varying periods of TGF-β1 treatment before treatment with EGF for 15 h. This revealed increased cooperation between TGF-β1 and EGF in the induction of *Hmga2* upon extended exposure to TGF-β1 (Figure 2B). The stronger EGF responsiveness on *Hmga2* corresponded well with elevated *Egfr* expression in response to TGF-β1, which peaked at 24 h. In addition, TGF-β1 also induced a delayed kinetics on *Lrig1*, which is a negative regulator of EGFR signaling important to the maintenance of gastrointestinal stem cells [12], [21]. The induction of *Lrig1* is concordant with the diminished EGF-responsiveness of *Hmga2* observed at later time points [21]. Consistent with the earlier observation, the cooperation between TGF-β1 and EGF was absent for *Snai1* and *Fn1*, two well-defined markers of TGF-β/Smad-induced EMT (Figure 2B). Therefore, these data suggest that the TGF-β1 pathway employs the EGF pathway to specifically induce stemness and plasticity, but not EMT.

To further characterize the contribution of EGFR signaling to cellular plasticity, resting and TGF-β1-activated GIF-14 cells were treated with inhibitors of TGF-βRI (SB431542), EGFR (AG1478) and MEK1/2 (U0126), which is a major effector of the EGFR/Ras pathway. The treatment of all three inhibitors resulted in a strong blockade of TGF-β1 induction of *Hmga2*, confirming the participation of EGFR pathway, in particular the Ras/MEK signaling axis (Figure 2C). Furthermore, these inhibitors greatly suppressed basal expression levels of *Hmga2* and *Lgr5*, revealing a contribution of the EGFR pathway in the maintenance of cellular plasticity in GIF-14 cells (Figure 2C). In sharp contrast, the activation of *Snai1* by TGF-β1 treatment was inhibited only by SB431542, and not the inhibitors of the EGFR/MEK pathway (Figure 2C). These observations provide compelling evidence that the induction of stemness and plasticity by TGF-β1 is mediated through the EGFR/Ras/MEK signaling axis.

It has been reported that TGF-β can directly transactivate the EGFR to effect a change in ERK phosphorylation, which is a major downstream effect of Ras activation [25]. To determine if this direct mechanism is involved, the effects of TGF-β1 on the phosphorylation states of Egfr and Erk1/2 in the immediate time scale were determined by Western blotting. This revealed that TGF-β1 rapidly induced a rapid dephosphorylation of Erk1/2 within 15 min, which was recovered after 120 min (Figure 2D, top panel). This was markedly different to the effect of EGF (Figure 2D, bottom panel), which rapidly induced Erk1/2 phosphorylation. A further difference could be observed in the induction of phosphorylated Egfr^Y1068^ and Egfr^Y1092^ by EGF, which is absent in the case of TGF-β1 treatment. Taken together, these data suggest that the transactivating activity of TGF-β1 on the EGFR pathway does not account for the observed induction of the EGFR/Ras gene expression signature in GIF-14 cells. Instead, it was likely mediated via secondary events, such as the induction of Egfr expression (Figure 1F and 2B).

### Activation of Ras in GIF-14 induces a stem cell-like state but not EMT

**Figure 3:**
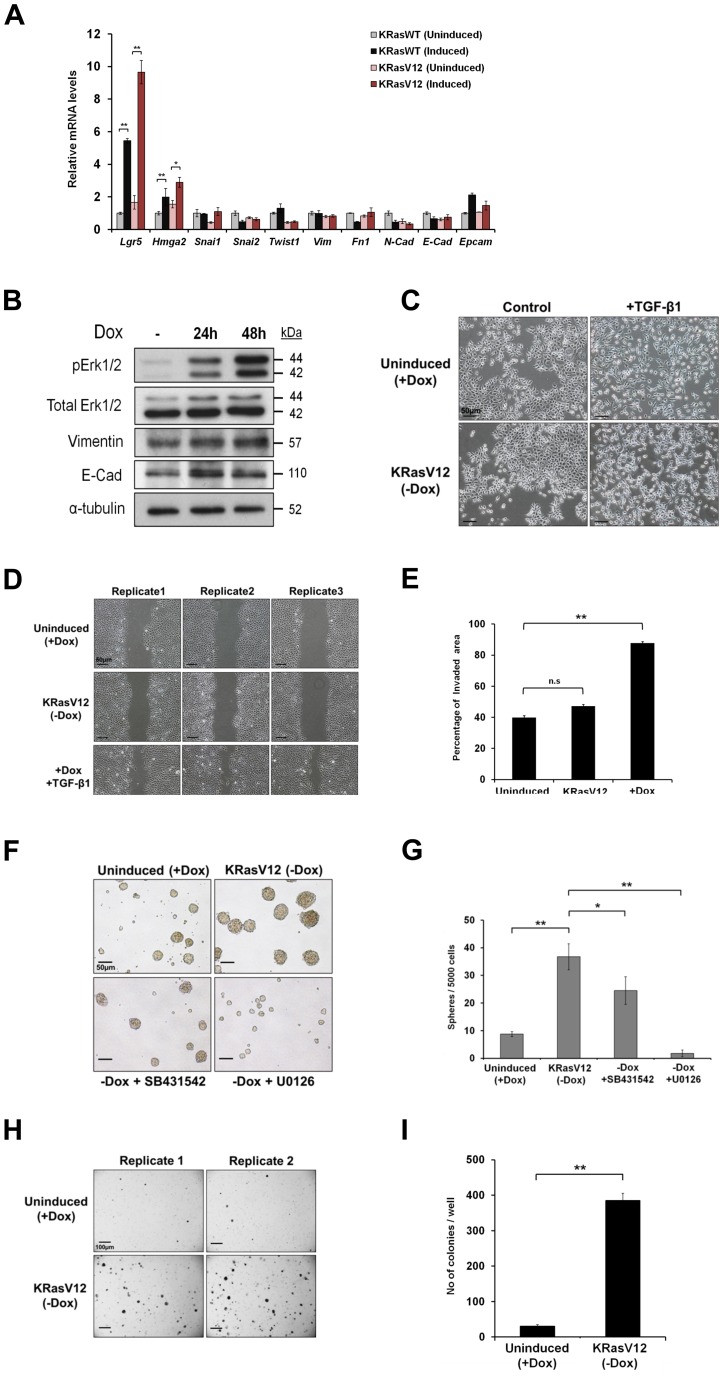
KRas robustly induces stemness and tumorigenicity in GIF-14 cells without triggering EMT. (A) Wild type (KRasWT) or oncogenic (KRasV12) KRas were induced for 48 h in pooled GIF-14/KRasWT and GIF-14/KRasV12 cells by the withdrawal of doxycycline (100 ng/ml). Expression levels EMT/mesenchymal and stemness markers were determined by qRT-PCR, normalized against *Gapdh* levels and presented relative to those of KRasWT uninduced sample (means ± SEM, n = 3). Student's t-tests are performed where indicated. Single and double asterisks denote *p* value<0.05 and *p* value<0.01, respectively. (B) The effects of KRasV12 activation on the expression of mesenchymal and epithelial markers. Changes in the expression levels of mesenchymal marker, Vimentin and epithelial marker, E-cadherin (E-Cad) upon the withdrawal of doxycycline (100 ng/ml) for 24 h and 48 h in GIF-14/KRasV12 cells were measured by Western blot analysis. The expression levels of phosphorylated Erk1/2 were determined to indicate the activation of Ras signaling pathway. Immunoblotting of α-tubulin serves as a control for the amount of proteins loaded. (C) The activation of KRas has no discernable effect on cell morphology of GIF-14/KRasV12 cells 48 h after doxycycline (100 ng/ml) was withdrawn, compared with those treated with TGF-β1 (2.5 ng/ml). Phase contrast images were captured. Scale bars = 50 µm. (D) The activation of KRas does not significantly alter the migratory properties of GIF-14 cells. GIF-14/KRasV12 cells were concurrently induced for KRas expression and co-treated with TGF-β1 (2.5 ng/ml) or carrier control for 24 h before the creation of scratch wounds. Phase contrast images were captured at 0 h and 12 h and representative pictures 12 h post-wounding are shown. Scale bars = 50 µm. (E) Graphical representation of wound healing assay data as analysed by the Tscratch software. Results presented are compiled from four replicates (means ± SEM, n = 4). Student's t-tests are performed and double asterisks denote *p* value<0.01 while n.s represents not significant. (F) KRasV12 promotes sphere formation in GIF-14 cells. Sphere-forming potentials of GIF-14/KRasV12 cells were determined upon the activation of KRasV12 in the presence of SB431542 (10 µM) or U0126 (10 µM). Representative images of the spheres are shown. Scale bars = 50 µm. (G) Graphical presentation of sphere assay results. The number of spheres ≥250 µm in size were scored after 6 days (means ± SEM, n = 3). Student's t-tests are performed. Single and double asterisks denote *p* value<0.05 and *p* value<0.01, respectively, (H) Anchorage-independent growth of GIF-14/KRasV12 cells was ascertained by soft agar assay following the induction of KRasV12. Representative images of the colonies at the seeding density of 200,000 cells/well are shown. Scale bars = 100 µm. (I) Graphical presentation of colony counts of soft agar assays in which each replicate was seeded at 200,000 cells/well and cultured for 2 weeks (mean ± SEM, n = 3). Student's t-tests are performed and double asterisks denote *p*-value<0.01.

The activation of Ras is a major downstream event following the binding of EGF to its receptor. To further establish an involvement of the Ras pathway in EMT-induced stemness, inducible GIF-14 lines were generated in which the expression of wild type (KRasWT) or a constitutively active mutant form of KRas (KRasV12) could be induced by the withdrawal of doxycycline from the culture medium. Quantitative RT-PCR measurement of changes in gene expression showed that the expression of both wild type and mutant KRas in GIF-14 cells for 48 h led to the clear induction of *Hmga2*, as well as the gastrointestinal stem cell marker *Lgr5* (Figure 3A).

Concordant with earlier observations, the activation of the Ras pathway did not alter the expression of relevant EMT markers in GIF-14 cells (Figure 3A). This is supported by Western blotting analysis, which showed that the expression level of Vimentin remained unchanged following the expression of oncogenic KRasV12 (Figure 3B). Similarly, the expression level of E-cadherin (E-Cad) was maintained at a consistent level, in keeping with the mRNA expression pattern (Figure 3B and 3A). Furthermore, the induction of KRasV12 did not significantly alter cell morphologies, compared with the induction of fibroblast-like morphologies by TGF-β1, which is a hallmark of EMT (Figure 3C). Lastly, the induction of KRasV12 did not significantly increase the migratory properties of GIF-14 cells in wound healing assays (Figure 3D and E). Together, these data suggest that the Ras pathway act to specifically promote stemness/plasticity without affecting the epithelial-mesenchymal states of GIF-14 cells.

### Activation of Ras pathway functionally promotes sphere formation and tumorigenicity

To functionally assay Ras-induced stemness and plasticity, GIF-14/KRasV12 cells were subjected to serum-free sphere culture in low attachment tissue culture substratum. The importance of the EGFR signal in this assay has been previously established in that exogenous EGF is necessary for the generation of GIF-14 spheres (data not shown). Accordingly, the activation of the Ras pathway in GIF-14/KRasV12 cells following the withdrawal of doxycycline significantly promoted sphere formation and growth. These enhancements were completely abolished with the blocking of MEK1/2 by U0126 (Figure 3F & 3G), consistent with the efficacy of this inhibitor in inhibiting MEK1/2 (Figure S2). Indeed, treatment with U0126 reduced sphere initiation to levels lower than that of the control (uninduced) sample, attesting to the contribution from the exogenous EGF in the culture medium (Figure 3F & 3G). Interestingly, despite the absence of exogenous TGF-β1 in the sphere culture, SB431542 partially reduced the effect of Ras, suggesting the involvement of autocrinal TGF-β in sphere formation (Figure 3F & 3G).

It was shown that the sphere-forming population in the parental GIF-14 line was also the tumorigenic fraction [18]. Therefore, GIF-14/KRasV12 cells were cultured in soft agar to measure anchorage-independent growth, an indicator of tumorigenicity. Consistent with the increase in stemness, a strong increase in anchorage-independent growth following the induction of KRasV12 was observed in these cells when plated in two different densities (Figure 3H & 3I; data not show). Together, these results indicate that the Ras pathway plays a significant role in promoting stemness and tumorigenicity in GIF-14 cells.

## Discussion

In recent years, the phenomenon of somatic reprogramming and induced pluripotency has shed new light into the plasticity and dedifferentiation associated with cancer [26], [27]. Similarly, the passage of differentiated cells through an EMT induces phenotypically plastic and multipotent “cancer stem cells”. These observations hold implications on the cell-of-origin of cancer [28]. Indeed, experimental evidence suggests that in addition to its role in cancer progression, EMT-induced plasticity promotes tumor initiation [29]. Therefore, understanding the factors that afford such plasticity, particularly the paracrinal signals involved in the maintenance of normal and cancer stem cells, is of interest.

The loss of the tumor suppressor gene *Runx3* is an early event during gastric carcinogenesis [19], [20]. In a previous study, immortalized *Runx3* ^−/−^ *p53* ^−/−^ gastric epithelial cells that have undergone spontaneous EMT was reported to be endowed with stem-like and mesenchymal properties and increased tumorigenicity [18]. The increase in cellular plasticity was reflected in a mesenchymal-like state and the reactivation of a number of stem cell markers, most notably *Hmga2* and *Lgr5*. However, the delayed kinetics of their reactivation suggested the involvement of intermediate events. In elucidating the signaling pathway underlying this phenomenon, the current study reveals that TGF-β1 induces stemness and plasticity by utilizing the EGFR/Ras signaling pathway, with the specific activation of *Hmga2*, a non-histone component of the chromatin associated with stemness, plasticity and tumorigenicity [22]–[24], [30], [31]. Treatment of pre-EMT GIF-14 cells with TGF-β1 triggered an EGFR/Ras gene expression signature that arose concurrently with the initiation of EMT. This phenomenon corresponded with an induction of Egfr expression that sensitized GIF-14 cells to the induction of *Hmga2* by EGF.

**Figure 4:**
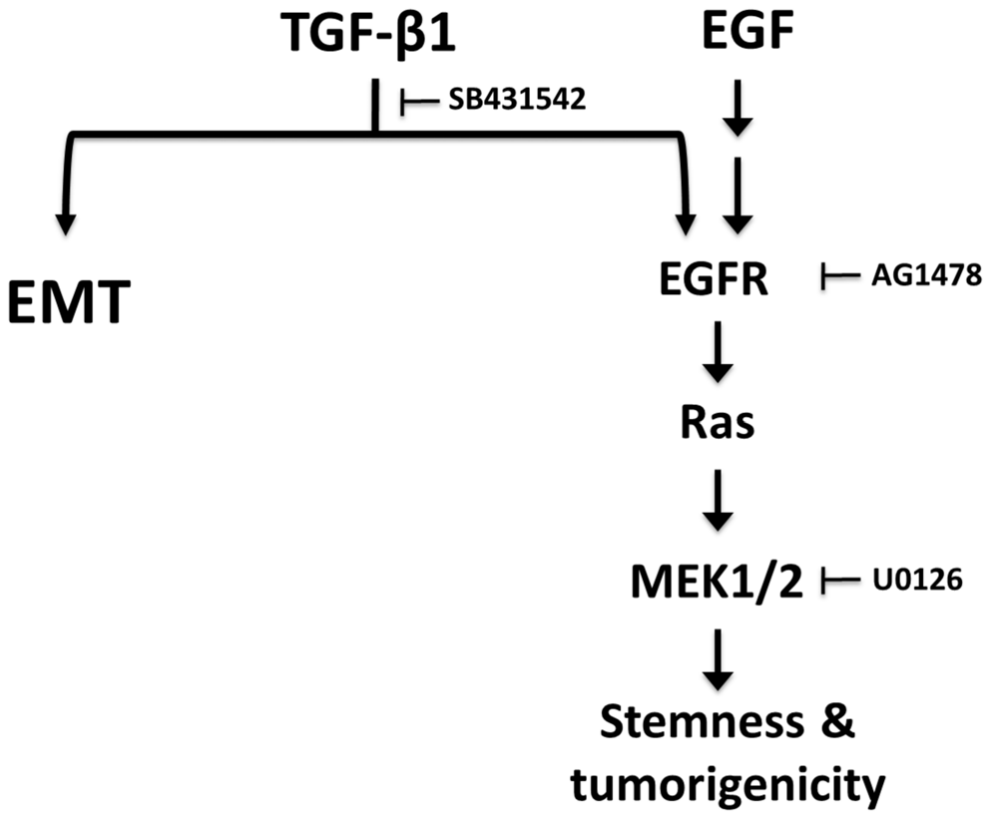
Proposed model for the contribution of EGFR/Ras/MEK signaling pathway to TGF-β1-induced stemness and tumorigenicity. The activation of TGF-β pathway triggers *Runx3^−/−^p53^−/−^* GIF-14 gastric epithelial cells into EMT, concurrently inducing a stem cell-like and tumorigenic state. The data presented in this report show that TGF-β1 induces an EGFR/Ras gene expression signature marked by increased Egfr expression, which sensitized GIF-14 cells to EGF. The activation of EGFR/Ras pathway promotes stemness and tumorigenicity in GIF-14 cells in a MEK1/2-dependent manner that did not involve increased EMT.

TGF-β was originally described and named for its ability to mediate the phenotypic transformation of normal rat kidney fibroblast [32]. This property was subsequently found to be dependent on the activation of EGFR by TGF-α or EGF [33], [34]. Although the engagement of EGFR to its ligands is known to activate several divergent downstream pathways, the activation of *Hmga2* by TGF-β1 in the GIF-14 cell model is effectively blocked by a pharmacological inhibitor to MEK1/2, indicating the participation of the Ras-MEK signaling axis. This involvement was confirmed in the induction of *Hmga2* and *Lgr5* by exogenous wild type or oncogenic KRas in GIF-14/KRasWT and GIF-14/KRasV12 inducible lines, respectively. Importantly, the EGFR/Ras/MEK signal functionally supported in GIF-14 cells the generation of spheres, an *in vitro* assay of stemness; as well as increased colony formation in soft agar assay, a classical assay of cellular transformation and tumorigenicity. Together, these data support a model in which TGF-β1-induced EMT is accompanied by an activation of the EGFR/Ras/MEK signaling pathway via increased Egfr expression; which in turn drives a stem-like and tumorigenic state in GIF-14 cells, as depicted in Figure 4. While the data presented in this study were derived from *in vitro* experiments, the *in vivo* relevance of this relationship could be appreciated in the recent report that the targeting of *Lrig1*, a negative regulator of Egf/ErbB receptor signaling, in a *Lrig1* ^+ve^ stem cell population resulted in increased Egfr and Lgr5 expression *in vivo* and intestinal tumors [12].

In addition to *Egfr*, the EGFR/Ras expression signature induced by TGF-β1 in GIF-14 cells consisted of other positive and negative regulators of the EGFR/Ras pathway, such as *KRas* and *Lrig1*, respectively. It is likely that TGF-β1 activates an EGFR/Ras expression program in GIF-14 cells *en route* to the maintenance of a metastable stem cell-like state. This is reminiscent to the reported cooperation between TGF-β and Wnt pathways in GIF-14 cells that synergistically induced *Lgr5*, a strong amplifier of the Wnt signal that supports normal and cancer stem cells [8], [12], [16]–[18], [35]. These observations suggest that TGF-β, in addition to the phenotypic plasticity that it endows via EMT, activates a series of genetic programs to promote a stem cell-like state. It will be of great interest to determine if this pathway to cellular plasticity and tumorigenicity involves additional signal downstream of TGF-β1, EGFR and Wnt signaling; and if the genetic circuits activated by these pathways are self-sustaining and mutually enforcing.

In GIF-14 cells, the passage through TGF-β1-induced EMT is marked by an acquisition of stem cell-like property, a process that is hitherto tightly coupled to the induction of mesenchymal and EMT-associated marker genes [18]. A key observation of the current study is that KRas-induced stemness was not accompanied by EMT, as reflected in an unaltered EMT/mesenchymal gene expression signature. This revealed an unexpected uncoupling in the induction of stemness and EMT in GIF-14 cells, providing evidence that they are driven by distinct, though coordinated cellular signals. Although the EGFR/Ras pathway is known to promote EMT-like phenotypes in mammary epithelial cells [4], [36]; hepatocytes [37], [38] and lung epithelial cells [39], the insufficiency of KRas to trigger an EMT in GIF-14 cells suggests that other signaling modules downstream of TGF-β1 are requisite. This is supported by the effective abrogation of EMT by a TGF-βR1 inhibitor (data not shown).

Lastly, EMT-induced stemness and tumorigenicity is a property restricted to *Runx3* ^−/−^ *p53* ^−/−^ fetal gastric epithelial lines and not their *Runx3* ^+/+^ *p53* ^−/−^ counterparts, which reflects a safeguarding function of Runx3 against cellular plasticity [18], [19], [40]. Consistent with our observation that *Hmga2* is a marker of TGF-β1/EGF-induced plasticity in GIF-14 cells, a recent study reports a surprising activation of a latent gastric differentiation program during KRas-driven lung tumorigenesis that is marked by the expression of Hmga2 [24]. In the same model, tissue-specific ablation of *Runx3* in lung epithelial cells greatly accelerated KRasV12-driven lung carcinogenesis (SC Bae, *pers comm.*). Collectively, these observations suggest that Runx3 serves as an important tumor suppressor against Ras-induced tumorigenicity by safeguarding against Hmga2-mediated plasticity and stemness. Therefore, it will be of future interest to determine the contribution of Runx3 in suppressing EGFR/Ras-mediated stemness and tumorigenicity in GIF-14 cells.

## Materials and Methods

### Cell culture and treatment

The murine gastric epithelial cell line GIF-14 was established from *Runx3* ^−/−^ *p53* ^−/−^ fetal mouse stomachs [Fukamachi, 2004]. These cells were maintained in Dulbecco's Modified Eagle Medium (DMEM; Invitrogen, CA, USA) with 4500 mg/l glucose supplemented with 10% FBS, 100 units/ml penicillin and 100 µg/ml streptomycin (Thermo Scientific Hyclone, UT, USA), in a 5% CO_2_ incubator at 37°C. To activate various growth receptor pathways, GIF-14 cells were treated with recombinant murine epidermal growth factor (EGF; 10 ng/ml; PeproTech, NJ, USA); human EGF (10 ng/ml; PeproTech), fibroblast growth factor (FGF)-2 (10 ng/ml; PeproTech); FGF-10 (10 ng/ml; PeproTech), hepatocyte growth factor (10 ng/ml; a gift from Jean Paul Thiery) and transforming growth factor-beta 1 (TGF-β1; 2.5 ng/ml; R&D Systems, MN, USA) for the time periods indicated. Inhibition of TGF-βRI, EGFR and MEK1/2 were achieved by treatment with SB431542 (10 µM in DMSO; TOCRIS Bioscience, Bristol, UK), AG1478 (10 µM in DMSO; TOCRIS Biosciences) and U0126 (10 µM in DMSO; TOCRIS Biosciences), respectively.

### Expression microarray analysis

Pre-EMT GIF-14 cells were treated with 2.5 ng/ml of recombinant TGF-β1 (R&D Systems) for 24 h. Total RNA was extracted from biological triplicates of TGF-β1-treated and untreated cells using QIAGEN RNeasy Mini Kit (QIAGEN, Hilden, Germany). Illumina TotalPrep RNA Amplification Kit (Ambion, TX, USA) was utilized to amplify and biotin-label 500 ng of RNA prior hybridization to MouseRef-8 Gene Expression BeadChip version 2 (Illumina, CA, USA). The raw data was pre-processed by Illumina GenomeStudio Gene Expression module version 1.9 using quantile and average normalization methods with default parameter settings. The data was log2 transformed for downstream analysis. Statistical Analysis of Microarrays (SAM) was applied on the normalized data to identify differentially expressed genes (q-value<0.05) [41]. To identify pathways enriched in TGF-β1-treated cells, Gene Set Enrichment Analysis (GSEA) was performed and pathways with nominal p-values smaller than 0.05 were selected [42]. Log-average expression of leading-edge genes was utilized to compute the sample enrichment scores of various pathways to the microarray data [43]. Raw microarray data have been deposited to the Gene Expression Omnibus (Accession Number GSE44055).

### Gene expression profiling by quantitative RT-PCR

Parental or derived lines of GIF-14 cells were cultured and treated in 48-well plates. RNA extraction was performed using the iScript RT-qPCR Sample Preparation Reagent (Bio-Rad, CA, USA). The syntheses of cDNA were performed with the iScript Reverse Transcription Supermix for RT-qPCR (Bio-Rad). Quantitative PCR was performed with gene-specific oligonucleotide primers using SsoFast EvaGreen Supermix (Biorad) or Kapa SYBR Fast Universal qPCR Master Mix (Kapa Biosystems, MA, USA); or with Taqman probes (Applied Biosystems, CA, USA) using SsoFast Probes Supermix (Bio-Rad) on a Applied Biosystems 7500 real-time PCR system (Applied Biosystems). A complete list of the oligonucleotide primer sequences and Taqman probes used are provided in Table 1. Unless otherwise stated, all gene expression data were normalized against *Gapdh* levels.

**Table 1:** Oligonucleotide primers and Taqman probes used for quantitative RT-PCR.

SYBR green primers		
Gene	Forward primer (5′-3′)	Reverse primer (5′-3′)
*E-Cad*	GAGCGTGCCCCAGTATCG	CTGCCTTCAGGTTTTCATCGA
*Egfr*	GGAAGTATGCAGATGCCAATAATG	GGCCCAGCACATCCATAGG
*Epcam*	ACGGAGAGCCGCTCGAT	GGGTGCCTTTTCATCAACGT
*Fn1*	CATGCCTCGGGAATGGAA	TGCCACTGTTCTCCTACATGGT
*Hmga2*	AGCTTGTTTGGTTTTCAGTGTCTTT	GTATGGACAAGAGGAATTACAGGAAGAG
*Lgr5*	TTCAATCCCTGCGCCTAGAT	TGCAGGCCGCTGAAACA
*Lrig1*	GGATTTTCCAATAGTGAGGGTTAGC	CCAGTCTCACCGGTAAGGAACA
*N-Cad*	GCCATCATCGCTATCCTTCTG	CGCCGTTTCATCCATACCA
*Snai1*	CTGCAACCGTGCTTTTGCT	CACATCCGAGTGGGTTTGG
*Snai2*	ACACAGTTATTATTTCCCCATATCTCTATGA	CCGACGATGTCCATACAGTAATAGG
*Twist1*	CACGCAGTCGCTGAACGA	GACCTGGTACAGGAAGTCGATGT
*Vim*	CCTGAGAGAAACTAACCTGGAGTCA	CATCTCTGGTCTCAACCGTCTTAA

TaqMan probes	
Gene	Taqman ID
*Gapdh*	Mm99999915_g1

### Soft agar colony assay

GIF-14/KRasV12 cells were suspended in 0.4% agarose and DMEM supplemented with 10% FBS and seeded over a basal layer of 0.6% agarose. The experiments were set up in 6-well plates at cell densities of 200,000 and 500,000 cells/well in triplicates. Colonies were scored manually after 2 weeks of culture at 37°C. Phase contrast micrographs of the colonies were captured on an Olympus SZX12 microscope (Olympus, Tokyo, Japan).

### Sphere-forming assay

GIF-14/KRasV12 cells were seeded at a density of 5000 cells/well in 6-well ultra-low attachment plates (Sigma-Aldrich, MO, USA) and cultured in serum-free DMEM:F12 medium (Invitrogen) containing 20 ng/ml human recombinant EGF (PeproTech), 10 ng/ml human recombinant FGF-2 (PeproTech), B27 (Invitrogen), N2 (Invitrogen), 1 ng/ml hydrocortisone (StemCell Technologies, CA, USA), 5 ug/ml insulin (Invitrogen) and 0.4% BSA Fraction V (Sigma-Aldrich). Methylcellulose (Sigma-Aldrich) was added to prevent cell aggregation to a final concentration of 0.5%, unless otherwise stated. At the indicated time, the number of spheres of the indicated sizes were counted and imaged under phase contrast microscopy performed on a Nikon Eclipse TS100 (Nikon, Tokyo, Japan).

### Virus production and transduction

The pRevTRE-KRas and pRevTre-KRasV12 and pBABE-puro-tTA (Tet-off) retroviral constructs were kindly provided by Drs. Matthias Drosten and Mariano Barbacid (CNIO; Madrid, Spain) [44]. pRev- and pBABE-based transfer vectors were co-transfected with the ecotropic helper retrovirus plasmid pCL-Eco (a gift from Motomi Osato) into HEK 293T cells using FuGENE HD (Roche, Basel, Switzerland). Supernatants containing viral particles were harvested 36 h and 48 h post-transfection and pooled. For transduction with retroviruses, GIF-14 cells were incubated with virus-containing supernatants in the presence of 5 µg/ml polybrene (Sigma-Aldrich) for 24 h before replenishment with normal culture medium. Cells were then subjected to puromycin (5 µg/ml; Invitrogen) or/and hygromycin (800 µg/ml; Invitrogen) selection and maintained in the presence of doxycycline (100 ng/ml; Clontech, CA, USA). Wild type and the oncogenic form of KRas were activated for 24 or 48 h by doxycycline withdrawal prior to direct lysis for RNA extraction and gene expression profiling.

### Western blot analysis

GIF-14 cells treated with TGF-β1 or EGF for the indicated time periods or GIF-14/KRasV12 cells were harvested for Western blot analysis. Cell pellets were lysed by RIPA buffer (50 mM Tris-HCl pH 7.4, 150 mM NaCl, 1% NP-40, 0.25% sodium deoxycholate and 1 mM EDTA) and whole cell lysates were resolved in 6% or 10% SDS-polyacrylamide gels. Murine total Egfr was detected using two separate anti-Egfr antibodies – Ab2430 (1∶200 dilution; Abcam, Cambridge, UK; a gift from Amy Lau Yong Chen and Peter Lobie) and #2232 (1∶1000 dilution; Cell Signaling, MA, USA; a gift from Manoj Garg and H. Phillip Koeffler). Immunoblotting with anti-phosphorylated Egfr (Egfr^Y1068^ and Egfr^Y1092^; 1∶1000 dilution; Abcam), anti-Erk1/2 (1∶1000 dilution; BD Transduction Laboratories, NJ, USA), anti-phosphorylated Erk1/2 (1∶1000 dilution; Cell Signaling), anti-E-Cadherin (1∶1000 dilution; Abcam), anti-Vimentin (1∶500 dilution; Abcam), anti-α-tubulin (1∶10000 dilution; Sigma-Aldrich), anti-mouse IgG-HRP (1∶3000 dilution; GE Healthcare, Buckinghamshire, UK), anti-Rabbit IgG HRP (1∶3000 dilution; GE Healthcare) and anti-rat IgG HRP (1∶3000 dilution; Dako, Glostrup, Denmark) antibodies were performed.

### Wound healing assay

KRasV12 was activated in GIF-14/KRasV12 cells and concurrent treatment with TGF-β1 for 24 h was performed. Cells were grown to confluence in 6-cm dishes and wounds were generated using 200 p pipette tips. Phase contrast images were captured at 0 h and 12 h. For each sample, 4 images were taken from 3 wounds and the imaging data was analysed by the Tscratch software [45].

### Densitometric analysis

The intensities of protein bands from Western blots were determined using Adobe Photoshop CS3 software. Three different samplings were performed and the band intensities were normalized against those of α-tubulin.

### Statistical analysis

All data are presented as the means ± SEM. Student's t-test was utilized in the comparisons of two data sets, and *p* value<0.05 were considered significant.

## Supporting Information

Figure S1 **TGF-β1 and EGF cooperate to induce** ***Hmga2*** **in GIF-14 cells.** The effects of various growth factor treatment on the gene expression of stemness- and EMT/mesenchymal-associated markers. GIF-14 cells were treated with murine EGF (mEGF; 10 ng/ml), human EGF (hEGF; 10 ng/ml), FGF2 (10 ng/ml), FGF10 (10 ng/ml) or HGF (10 ng/ml) in isolation or in combination with TGF-β1 (2.5 ng/ml) for 24 h. Changes in the gene expression levels were measured by qRT-PCR. The values are normalized against *Gapdh* levels and expressed relative to the control sample.
(TIF)

Figure S2 **Phosphorylation of Erk is blocked by a MEK1/2 inhibitor in TGF-β1- or KRasV12-activated cells.** GIF-14 cells were treated with TGF-β1 (2.5 ng/ml) while GIF-14/KRasV12 cells were induced for KRasV12 activation in the presence of U0126 (10 µM) for 24 h and 48 h. The expression levels of phosphorylated Erk1/2 were assessed by Western blot analysis and immunoblots of α-tubulin is used as a control for the amount of proteins loaded.
(TIF)
