# Supplementary figures and images for: EMT-Induced Stemness and Tumorigenicity Are Fueled by the EGFR/Ras Pathway
Source: PLoS One. 2013 Aug 12;8(8):e70427. doi: 10.1371/journal.pone.0070427 (PMC3741305; doi:10.1371/journal.pone.0070427)

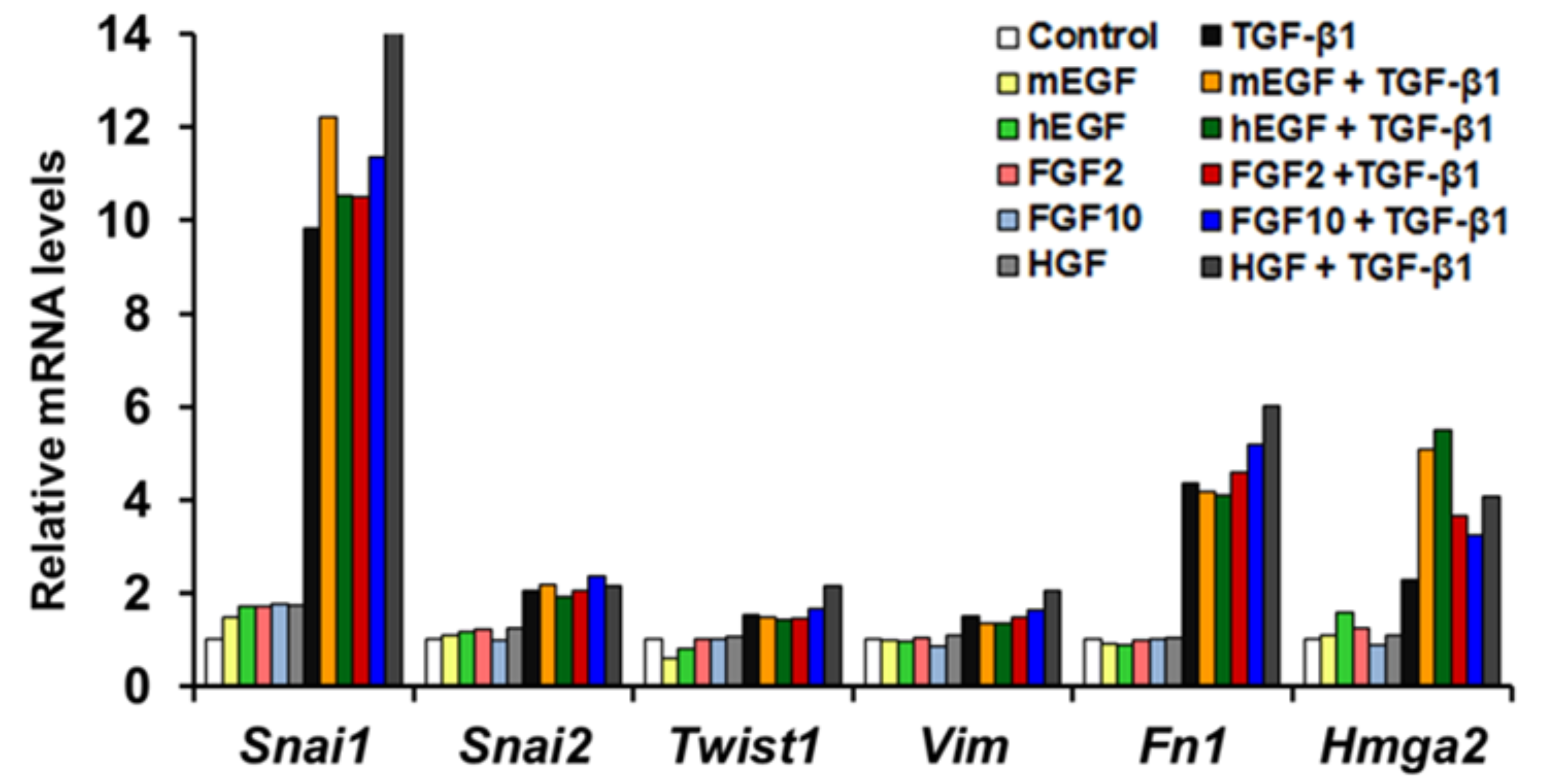

Supplement: Figure S1 — TGF-β1 and EGF cooperate to induce Hmga2 in GIF-14 cells. The effects of various growth factor treatment on the gene expression of stemness- and EMT/mesenchymal-associated markers. GIF-14 cells were treated with murine EGF (mEGF; 10 ng/ml), human EGF (hEGF; 10 ng/ml), FGF2 (10 ng/ml), FGF10 (10 ng/ml) or HGF (10 ng/ml) in isolation or in combination with TGF-β1 (2.5 ng/ml) for 24 h. Changes in the gene expression levels were measured by qRT-PCR. The values are normalized against Gapdh levels and expressed relative to the control sample. (TIF) [file pone.0070427.s001.tif]

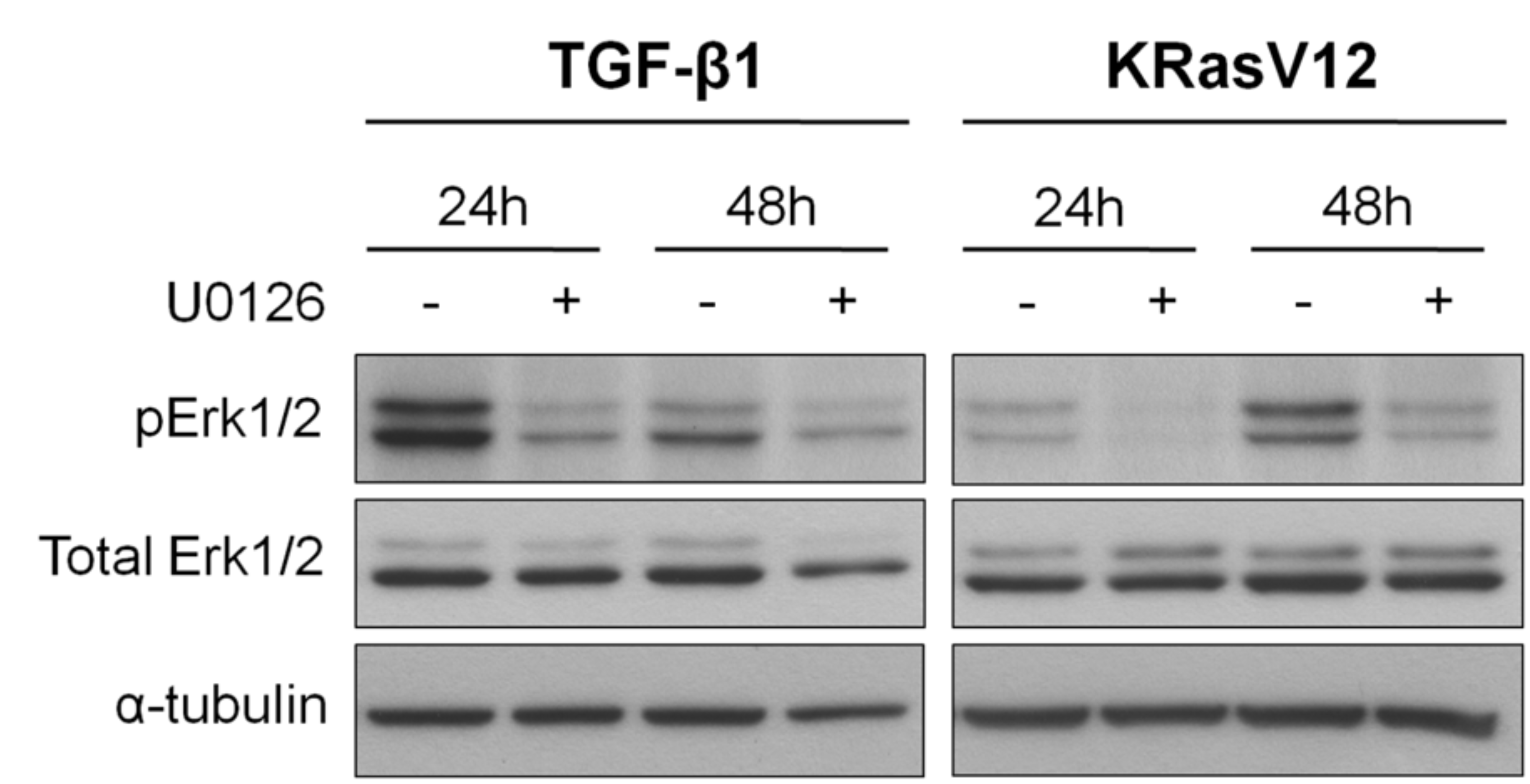

Supplement: Figure S2 — Phosphorylation of Erk is blocked by a MEK1/2 inhibitor in TGF-β1- or KRasV12-activated cells. GIF-14 cells were treated with TGF-β1 (2.5 ng/ml) while GIF-14/KRasV12 cells were induced for KRasV12 activation in the presence of U0126 (10 µM) for 24 h and 48 h. The expression levels of phosphorylated Erk1/2 were assessed by Western blot analysis and immunoblots of α-tubulin is used as a control for the amount of proteins loaded. (TIF) [file pone.0070427.s002.tif]
